# Evaluation of a Novel Pharmacist-Delivered Adherence Improvement Service via Telehealth

**DOI:** 10.3390/pharmacy9030140

**Published:** 2021-08-17

**Authors:** Srujitha Marupuru, Harman Dhatt, Jennifer M. Bingham, Terri Warholak

**Affiliations:** College of Pharmacy, University of Arizona, Tucson, AZ 85721, USA; dhatt@pharmacy.arizona.edu (H.D.); jbingham@trhc.com (J.M.B.); warholak@pharmacy.arizona.edu (T.W.)

**Keywords:** adherence, medication therapy management, pharmacist, telehealth

## Abstract

Nearly half of all patients prescribed a chronic medication do not adhere to their regimen. Conversion from a 30- to 90-day medication refill is associated with improved adherence. The objective of the study was to assess the change in proportion of days covered (PDC) in those who converted to a 90-day fill and those who did not after a telehealth pharmacist-delivered, medication adherence intervention. This retrospective review involved data collected between May and December 2018. Patients with ≤85% baseline PDC rates were targeted. One group included patients who converted to a 90-day fill after the pharmacist intervention. The comparator group did not convert to a 90-day fill. Differences in median end-of-year (EOY) PDC rates for each medication class were compared between groups. An alpha level of 0.05 was set a priori. Overall, 237 patients converted to a 90-day fill and 501 did not. There was no significant difference in age, sex, and total number of drugs per patient. A Mann–Whitney U test revealed statistically significant improvements in median EOY PDC in the group that converted to a 90-day fill (+9% vs. −3%, *p* < 0.001). Pharmacist-delivered telehealth interventions were associated with improved PDC rates in those who converted to a 90-day fill.

## 1. Introduction

Medication adherence is associated with improved clinical outcomes and reduced mortality, whereas non-adherence is associated with deteriorated health outcomes, higher hospitalization rates and increase in healthcare costs [1,2]. It is important to improve medication non-adherence, given its association with increased costs [3]. For managed care, pharmacist-delivered interventions are of particular importance to quality improvement (QI), as they can improve patient health outcomes and simultaneously reduce pharmacy costs [4].

Healthcare organizations have implemented numerous programs and services to improve medication adherence [5,6]. The proportion of days covered (PDC) is currently the preferred method to measure medication adherence. Key components of medication adherence QI programming are delivering indication-specific patient counseling and converting medication refill frequency to 90-day cycles. A study by Taitel et al. found that patients with a 90-day medication refill cycle had improved medication possession ratios (MPR) and significant cost savings [7]. Furthermore, conversion of chronic medication classes to a 90-day medication refill and patient enrollment in automatic refill programs is shown to improve medication adherence [8]. It has also been shown that conversion to a 90-day medication refill was associated with an increased supply in medication, reduced transportation costs, through fewer required pharmacy visits, and decreased out-of-pocket copays expenses [9].

Yet, with the preliminary evidence that such interventions may improve medication adherence, the utility of this intervention in a medication therapy management (MTM) telehealth setting is still unexplored. Hence, one national medication therapy management (MTM) provider implemented a pilot program to study the outcomes of pharmacist-driven interventions for 90-day medication refills for chronic medication classes. The objective of the study was to measure the change in PDC for patients who converted to a 90-day medication refill cycle and those who did not after a pharmacist-delivered, medication adherence intervention in the MTM telehealth setting. Secondary outcomes included an assessment of the change in PDC rates for those who converted to 90-day medication refills for each chronic maintenance medication class (e.g., oral antidiabetic, angiotensin-converting enzyme/angiotensin II receptor blockers, and/or hydroxymethylglutaryl-coenzyme A reductase inhibitors medications).

## 2. Materials and Methods

### 2.1. Program Description

A national MTM provider implemented an adherence improvement service in 2018 to promote conversion from 30- to 90-day medication refills using a pharmacist delivered telehealth model. The service allowed pharmacists to intervene on patients who had a PDC of <85% and who were eligible for a 30-day refill for any of the following chronic maintenance medication classes: oral antidiabetic agent, angiotensin-converting enzyme/angiotensin II receptor blocker (ACE/ARB), or hydroxymethylglutaryl-coenzyme A reductase inhibitors (statins). Pharmacists were employed at one of five national call centers and received standardized training on the provision of MTM care via synchronous audio-only telehealth delivery models. The patients included in this dataset were from a health plan which aimed to increase the PDC beyond the minimally acceptable value set by CMS measures; thus, PDC < 85% was used as the threshold for inclusion. MTM pharmacists used proprietary software, in tandem with real-time prescription claims data, to identify eligible Medicare patients for the service. Once the patient was identified through an alert prompted by the software, they were contacted by the pharmacist via telephone to complete a comprehensive medication review (CMR) and discuss conversion of their 30-day medication refill to a 90-day medication refill. The pharmacist also counseled the patient on methods to improve medication adherence, addressed barriers and provided a personalized medication list and letter to the patient through the mail with actionable items. If the patient consented to the 90-day medication refill conversion, the pharmacist formulated a recommendation that was sent via facsimile to the prescribing provider. If the patient did not refill a 90-day medication refill of their chronic medication within 60 days of the initial intervention, the pharmacist repeated the service offering.

### 2.2. Study Design

This retrospective review included data collected between May and December 2018. Participants were included in the study if they were continuously enrolled in the adherence improvement service (i.e., based on eligibility to receive an annual CMR as part of their Medicare benefits) and eligible for a refill of their oral antidiabetic agent, ACE/ARB, or statin prescription. Patients were excluded if they (1) were younger than 17 years, (2) were older than 89 years, (3) were non-Medicare beneficiaries, (4) had a baseline PDC rate of greater than 85% for one or more medication classes, (5) did not receive a CMR, or (6) were already receiving 90-day medication refills. This retrospective review was approved by the Institutional Review Board (approved on 18 September 2018, protocol No. 1809940982).

### 2.3. Data Collection

Data collected for each patient included demographics, total number of medications, PDC recorded at the time of the intervention, PDC at end-of-year (EOY) and presence of a 90-day medication refill within 60 days post-pharmacist intervention. Claims data were used by the national MTM provider to calculate PDC rates and those who were eligible for a refill. Medication adherence was measured using PDC rates and stratified by medication class. The PDC at the time of pharmacist intervention was used as the baseline rate. All patient data were deidentified, then provided by the national MTM provider to the research team.

### 2.4. Study Analysis

The primary outcome was the association of the 90-day medication refill conversion on PDC rates following a pharmacist adherence improvement intervention. Secondary outcomes included an investigation of the effect of 90-day medication refill conversion for all medication classes and exploring the differences in median EOY PDC rates for each chronic maintenance medication class. Statistical analysis was performed using a paired *t*-test and a Mann–Whitney U test. All comparisons were two-sided and used an a priori alpha level of 0.05. Data analyses were conducted using SAS version 9.1. (SAS Institute Inc., Cary, NC, USA).

## 3. Results

A total of 738 patients met the study inclusion criteria and received a pharmacist intervention. Of those, 237 (32%) patients were converted to a 90-day medication refill after receiving the national pharmacist-delivered adherence improvement service. A majority of these patients were female (58%), with a mean age of 68 ± 7 years. There were 501 (68%) patients that did not convert to a 90-day medication refill. A majority of these patients were female (60%), with a mean age of 69 ± 10 years. The mean number of medications per person was 13 ± 6 and 14 ± 7 in the converted to 90-day and did not convert to 90-day medication refill group, respectively. Hence, there was no significant difference in age, sex, or total number of drugs for those who converted to a 90-day medication refill and those who did not convert to a 90-day medication refill within 60 days of receiving the pharmacist intervention service. See Table 1 for a description of characteristics and a further breakdown of patients by alert type based on therapeutic area. There was no statistically significant difference between the percentage of participants who received each alert type.

A statistically significant difference in the median EOY PDC was observed between groups during the study time frame. Patients who converted to a 90-day medication refill had a greater improvement in their EOY median PDC rate compared to those who did not convert to a 90-day medication refill (79% vs. 70%, *p* < 0.001). Overall, they also had a greater difference in their median EOY PDC for all chronic maintenance medication classes (+9% in those who converted to 90 days vs. −3% in those who did not convert to 90 days, *p* < 0.001). See Table 2 for more detail.

Subgroup analyses by medication class, as shown in Table 3, showed a statistically significant difference between both groups in the median EOY PDC for oral antidiabetic agents, ACE/ARB and statin prescriptions. The improvements in median EOY PDC rates were observed in the group who converted to a 90-day medication refills for oral antidiabetic agents (16%), ACE/ARBs (12%) and statins (3%). The group that did convert to a 90-day medication refills had a larger absolute median increase in the EOY PDC rate compared to those who did not convert (oral antidiabetics, 16% vs. 5%; ACE/ARB, 12% vs. −1.50%; statin, 3% vs. −7%, respectively).

## 4. Discussion

The study results suggest that 90-day medication refill conversions of either an oral antidiabetic, ACE/ARB, or statin prescription facilitated by pharmacist interventions may improve medication adherence. Overall, there was an increase in the median EOY PDC rates among patients who converted to a 90-day medication refill. The positive association of the adherence intervention with the PDC rates was seen with all three medication classes examined.

Overall, medication adherence rates were marginally higher in patients who converted to a 90-day medication refill following the adherence intervention. These results align with the results of other studies [10,11,12]. For example, Leslie et al. performed an adherence intervention to convert Medicare beneficiaries’ 30-day medication fills to 90-day refills and found significantly improved medication adherence for patients prescribed an antihypertensive or statin medication [10]. Another study, which compared medication adherence for oral antidiabetic medications, hyperlipidemia agents and anti-hypertensive medications, found that patients who converted to a 90-day medication fill had mean unadjusted PDC rates significantly higher than those who did not convert to 90-day medication fills [11]. Similar results were also seen in patients who converted from 30-day to 90-day medication fills of medications for acute conditions, such as post-acute myocardial infarction with higher adherence one-year post discharge [12].

Research evidence supports the notion that pharmacists play a crucial role in improving adherence for chronic conditions. For example, a prospective quality-improvement project conducted by community pharmacists provided telephone interventions to improve adherence to hypertension and diabetes medications [13]. Patients having PDC rates less than 80% were identified, and telephonic interviews were conducted with recommendations such as patient education, medication synchronization program and the changing refill day supply from 30- to 90-days. Overall, 69% of patients with hypertension and 64.3% of patients with type 2 diabetes reached a PDC greater than 80% within 3 months [13]. Another collaborative pilot program with community pharmacists assessed targeted medications among Medicare members and a variety of adherence-based interventions [14]. Common interventions included explaining the benefit of the medication, provider follow-up and recommendations of adherence aids, such as pill boxes and changes to 90-days supplies. Post interventions mean PDCs increased by 14% (74–88%, *p* < 0.0001) [14].

In addition to adherence, there is evidence that converting to a 90-day medication fill has other benefits. One study found that changing to 90-day medication fills may help patients given its association with fewer refills and lower copays [14]. A white paper by Honeybee Health reported that among the most used ten maintenance medications, conversion to a 90-day medication fill significantly improved cost savings [15], while another white paper reported a 7% increase in cost savings for 90-day prescriptions compared to three 30-day fills, given decreased ingredient costs and dispensing fees [16]. In addition, there is some evidence that suggests generic utilization increases when 90-day prescriptions are filled [17].

Some barriers to 90-medication refills have been identified. According to published literature and anecdotal evidence, the greatest barrier to 90-day prescription implementation strategies are restrictions by prescription drug plans and payers [18]. Some commercial and Medicare drug plans limit the use of 90-day prescription refills of maintenance medications to retail and mail-order pharmacies [19]. More pervasive use of 90-day supplies would involve changing provider prescribing practices and increasing awareness about increased adherence associated with 90-day medication refills, both of which should be addressed in future provider education sessions and research. In addition, boosting consumer awareness of the 90-day medication refill option through store signage, web banners and mass media could be a successful outreach campaign to increase patient demand for 90-day refills [16].

### Limitations

This retrospective observational study had some limitations. For example, researchers were not able to measure actual medication taking behavior and the PDC results may have overestimated true adherence given the large sample size and small differences in PDC. While PDC is a validated claims-based adherence measure associated with health outcomes [20], researchers were not able to assess the clinical impact on chronic condition management nor the impact on other medication classes. Next, potential confounders were not controlled for, such as income, effects of structural racism, and the number of comorbidities. Further longitudinal studies should examine the normal fluctuation of medication adherence trends for all medication classes before and after the intervention. In addition, an assessment of patients with and without optimal baseline PDC rates would allow researchers to distinguish the impact of a 90-day refill conversion versus pharmacist telehealth services. Lastly, it would be prudent to assess the impact of the intervention on wastage and medication shortages.

## 5. Conclusions

Patients enrolled in an adherence improvement service who converted to a 90-day medication fill had greater improvements in end-of-year PDC rates for chronic maintenance medication classes than those who did not convert to a 90-day medication fill. Future longitudinal work is warranted to better understand patient-identified reasons for not converting to a 90-day refill and the impact of such interventions on clinical outcomes.

## Figures and Tables

**Table 1:** Patient characteristics.

Characteristics	Did Not Convert to 90-Day Refills (*n* = 501)	Converted to 90-Day Refills (*n* = 237)	*p*-Value ^2^
**Demographics**			
Age in years, mean ± SD	69 ± 10	68 ± 7	0.700
Sex, female, *n* (%)	260 (60)	137 (58)	0.133
Total drug count ^1^, mean ± SD	14 ± 7	13 ± 6	0.206
**Alert type by therapeutic area ^3^**			
ACE/ARB, *n* (%)	172 (34)	97 (41)	0.156
DM, *n* (%)	136 (27)	52 (22)	
Statin, *n* (%)	193 (39)	88 (37)	

^1^ Number of distinct drug trade names per claims. ^2^ *t*-test was used for continuous data and Pearson chi-square for categorical data to calculate *p*-value associated with each. ACE/ARB—angiotensin converting enzyme inhibitors/angiotensin-receptor blockers. DM—oral antidiabetic agents. ^3^ Medication class was categorized based on alert type specific to these disease conditions.

**Table 2:** Median proportion of days covered (PDC) and difference in PDC between groups.

Characteristics	Did Not Convert to 90-Day Refills (*n* = 501)	Converted to 90-Day Refills (*n* = 237)	*p*-Value ^2^
PDC at intervention (baseline) (%), median (IQR) ^1^	76 (66–81)	74 (65–80)	0.040
EOY PDC (%), median (IQR)	70 (44–83)	79 (65–88)	<0.001
Difference in EOY PDC rates ^3^ (%), median	−3	9	<0.001

PDC—proportion of days covered represented on a scale of 100%. IQR—interquartile range. EOY—end of year. ^1^ Median and interquartile range (IQR) is reported for PDC rates since PDC rates at alert trigger and end of year distribution are skewed. ^2^ Significance between groups was calculated using the Mann–Whitney U test. ^3^ Difference in median PDC rates in % was reported by SPSS using PROC UNIVARIATE function looking at the distribution of difference in EOY PDC rates and it is not a direct subtraction of “at intervention” and EOY. The PDC rates were calculated by the MTM provider.

**Table 3:** Median proportion of days covered (PDC) between groups categorized by medication class (subgroup analysis).

Med Class ^2^	Did Not Convert to 90-Day Refills (*n* = 501) Median (IQR), %				Converted to 90-Day Refills (*n* = 237) Median (IQR), %				*p* ^1^
	*n*	PDC at Intervention	EOY PDC	Difference in EOY PDC ^2^	*n*	PDC at Intervention	EOY PDC	Difference in EOY PDC ^2^	
ACE/ARB	172	76 (67–82)	73 (39–86)	−1.5	97	72 (62–80)	79 (62–89)	12	<0.001
DM	136	77 (67–81)	73 (50–86)	5	52	74 (62–80)	82 (59–93)	16	0.006
Statin	193	75 (65–80)	66 (44–79)	−7	88	76 (68–80)	78 (69–85)	3	<0.001

Med—medication. PDC—proportion of days covered. ACE—angiotensin-converting enzyme. ARB—angiotensin receptor blockers. DM—oral antidiabetic agents. IQR—interquartile range. EOY—end of year. ^1^ *p*-value was calculated using an independent samples Mann–Whitney U test to evaluate the difference in difference (change from PDC at alert trigger to the EOY PDC rates) between groups. ^2^ The difference in median PDC rates in % was reported by SPSS using PROC UNIVARIATE function looking at the distribution of difference in EOY PDC rates and it is not a direct subtraction of at intervention and EOY. Medication class was categorized based on alert type specific to these disease conditions.

## Data Availability

The data are not publicly available due to proprietary concerns.
